# AugGCL: Multimodal graph learning for spatial transcriptomics analysis with enhanced gene and morphological data

**DOI:** 10.1371/journal.pcbi.1013912

**Published:** 2026-01-23

**Authors:** Tengfei Ji, Bo Yang, Meng Wang, Hong Ji, Huazhe Yang, Yizhuo Liu

**Affiliations:** School of Computer Science, Xi’an Polytechnic University, Xi’an, Shaanxi, China; Allen Institute for Brain Science, UNITED STATES OF AMERICA

## Abstract

Spatial transcriptomics enables the measurement of gene expression in intact tissues. Despite this, reconstructing anatomically accurate spatial domains remains challenging, primarily due to expression sparsity, complex tissue architecture that is characterized by sharp boundaries and long-range continuity, and weak spatial signals. Traditional pipelines typically rely on expression-driven clustering and spatial smoothing, which underperform at boundaries and in sparse regions while neglecting morphological information. To address these challenges, AugGCL is proposed, an augmented graph-convolutional learning framework that enhances spatial structure decoding and gene expression reconstruction through targeted augmentation of both gene and image data. A key component of AugGCL is neighborhood information aggregation mechanism, which integrates expression similarity and spatial proximity to construct a weighted graph and an enhanced expression matrix, addressing sparsity without sacrificing boundary clarity. Additionally, a two stream weighted graph convolutional network jointly models refined gene features and image-derived morphological information, with image-aware auxiliary reconstructions enhancing weak spatial signals and sharpening boundaries. On datasets from the human dorsolateral prefrontal cortex, breast cancer, and mouse embryo, AugGCL outperforms baseline methods across multiple metrics, showing robustness and generalization across a range of datasets. Downstream analysis validated the reliability of the method, confirming its effectiveness in cell annotation, functional enrichment, and mechanistic studies. AugGCL generates clearer spatial domains and significantly advances the application of spatial transcriptomics in tissue structure and disease research.

## Author summary

Spatial transcriptomics is an important technique for revealing tissue structure and disease mechanisms. However, existing spatial domain identification methods have not fully exploited the spatial information embedded in the data, especially when it comes to detailed exploration of tissue structure. This study presents a new tool for spatial domain identification, which makes full use of various types of spatial transcriptomic data from multiple perspectives to accurately identify real cell groupings. The method significantly improves spatial domain recognition accuracy by integrating gene expression with tissue morphology analysis. Experimental results show that this tool not only accurately identifies spatial domains but also provides strong support for the comprehensive exploration of biological tissue structure. On this basis, a series of downstream analyses, including volcano plot generation, functional enrichment analysis, and gene heatmap visualization, were performed. These analyses not only validated the effectiveness of the method but also revealed the functional characteristics and expression patterns of cells within spatial domains. This step further confirmed the broad application potential of the method in cell type annotation, functional enrichment, and mechanistic research, highlighting its significant potential in advancing biological and disease research.

## Introduction

Recent advancements in spatial molecular imaging have opened new avenues for studying tissue architecture and gene expression at the subcellular level [1–3]. Deeper insight into cellular interactions within their microenvironment is crucial for elucidating disease mechanisms. Leading technologies such as 10× Visium and Vizgen MERSCOPE/MERFISH [4,5] are highly effective in capturing the spatial context of transcripts, cellular positioning, and boundaries, often integrated with high-resolution multi-channel immunohistochemistry (IHC) imaging. As these spatial transcriptomics (ST) tools continue to evolve, they are reshaping our approach to spatial biology, offering unparalleled insights into tissue organization and the molecular pathways underlying diseases [6–8].

Accurately identifying spatial domains using multimodal data remains a significant challenge in spatial transcriptomics research. Traditional spatial domain identification methods, such as Seurat [9] and Louvain-based Scanpy [10], rely solely on gene expression data, which often leads to suboptimal clustering results. In contrast, spatial clustering methods that incorporate spatial information and tissue image features have shown enhanced clustering performance. For example, BayesSpace [11] utilizes Bayesian statistics to analyze both gene expression matrices and spatial neighborhood information, achieving effective spatial clustering. Similarly, DeepST [12] improves correlations between spatially adjacent points by early fusion of gene expression data and tissue image features, leading to richer latent representations of spatial transcriptomics data. SiGra [13] applies an image-enhanced graph transformer model to analyze single-cell spatial data, which decodes spatial domains while amplifying spatial signals.

Despite the integration of tissue image features in these methods to enhance spatial gene expression representations and improve domain identification, gene expression matrices often suffer from sparsity [14–16], with many genes exhibiting very low or even zero expression in certain cells or regions. This issue is particularly prevalent in spatial transcriptomics datasets. Existing methods for addressing this sparsity include techniques such as imputation methods like MAGIC [17], spatial smoothing, and dimensionality reduction approaches. These methods aim to fill in missing data, reduce noise, and extract meaningful latent representations from sparse gene expression matrices. However, the sparsity and noise in gene expression data continue to significantly impact the latent information in spatial transcriptomics, making it a critical challenge for accurate spatial domain identification.

To address these challenges, the AugGCL method was developed, which uniquely tackles gene expression matrix sparsity while enhancing the identification of spatial domains. Unlike traditional methods that primarily rely on gene expression data, AugGCL integrates spatial neighborhood information with original gene features to create a smoother, more consistent spatial gene expression representation. By leveraging the spatial relationships between genes and cells, this method alleviates the expression void problem, which refers to regions of missing expression in gene expression matrices due to sparsity or noise. Additionally, the method uses graph convolutional networks (GCN) [18–20] to fuse multimodal data, including raw gene expression, enhanced gene expression, multi-channel tissue imaging, and cell spatial locations. By incorporating both gene and morphological information, AugGCL is able to capture complex spatial structures and dynamic tissue changes that traditional methods often overlook.

In contrast to existing methods, AugGCL addresses the inherent sparsity in gene expression matrices, improving the consistency and clarity of gene expression representations across spatial domains. Existing approaches, while effective in many cases, struggle to handle these sparsity issues, often leading to suboptimal clustering and low spatial resolution. AugGCL’s ability to fuse multimodal data and enhance the spatial consistency of gene expression maps makes it a more robust solution for identifying complex spatial domains and accurately modeling tissue structures.

Extensive tests across various ST datasets and benchmarks against existing algorithms demonstrate that AugGCL outperforms other methods in spatial domain identification, latent embedding, and data augmentation. AugGCL significantly improves clustering performance and spatial resolution, providing more biologically relevant and accurate spatial gene expression maps. AugGCL will play a key role in revealing the intricate spatial architecture within heterogeneous tissues, exploring cell-to-cell interactions, and advancing the field of spatial transcriptomics.

## Results

### Overview of AugGCL

**Fig 1:**
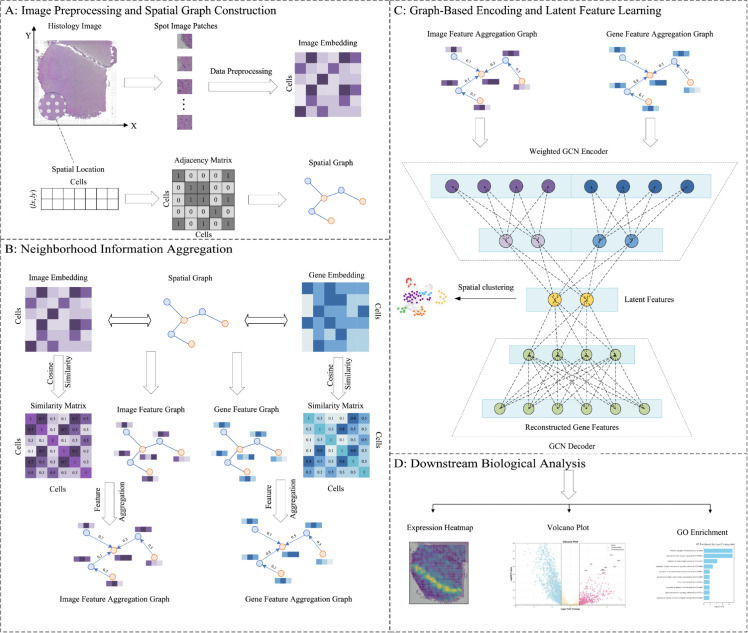
Illustrates the AugGCL-based spatial transcriptomics pipeline. (A) The data preprocessing and spatial graph construction steps are shown, where histological images are divided into patches, and spatial coordinates are used to create an adjacency matrix that captures cell proximity. (B) The neighborhood information aggregation process, where cosine similarity between cells is calculated based on both image and gene expression embeddings, constructing aggregation graphs with weighted edges. (C) The graph-based encoding and latent feature learning, where graph neural networks fuse the image and gene feature aggregation graphs to generate latent representations for each cell. These fused features are then used for spatial clustering and gene expression reconstruction, enabling a more accurate decoding of complex spatial structures. (D) The downstream biological analysis, where reconstructed gene expression matrices are used for spatial analyses such as heatmaps, volcano plots, and GO enrichment of upregulated genes, showcasing the biological relevance of the reconstructed data.

The AugGCL workflow (Fig 1) consists of several key stages, each corresponding to a specific component of the framework. First, data preprocessing filters out low-expression genes and generates an enhanced gene expression matrix, as shown in Fig 1A, where both data preprocessing and spatial graph construction take place. Spatial graph construction integrates gene expression and spatial adjacency data [21], laying the foundation for spatial analysis. Neighborhood Information Aggregation (NIA) then combines these elements to refine the expression matrix [22], improving spatial coherence. This step is illustrated in Fig 1B. The AugGCL model then fuses gene expression and tissue image features to improve spatial structure decoding, achieved through a multi-layer graph convolutional network, whose design is shown in Fig 1C. Finally, spatial clustering and biological analysis are conducted to interpret the represented results of data, yielding insights into cell interactions and molecular processes, as illustrated in Fig 1D.

### Spatial clustering of AugGCL on human dorsolateral prefrontal cortex tissue

To evaluate the clustering performance of AugGCL, it was compared with six commonly used spatial transcriptomics analysis methods: StLearn [23], SpaGCN [24], SEDR [25], GraphST [26], DeepST, and SiGra. All methods were applied to the human dorsolateral prefrontal cortex (DLPFC) dataset [27–30] obtained from the 10× Visium platform. This dataset contains 12 tissue slices, each annotated with six cortical layers (Layer 1–6) and white matter (WM).

**Fig 2:**
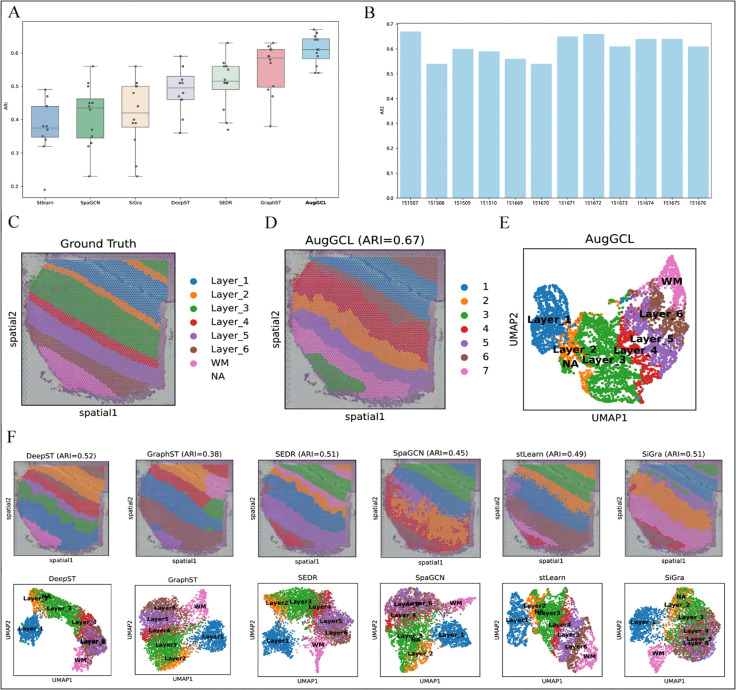
Comprehensive evaluation of spatial clustering models on DLPFC dataset. (A) ARI scores distribution of seven clustering models across 12 tissue slices. (B) ARI scores of AugGCL across individual samples, showing the stability of its performance across diverse tissues. (C) Ground truth annotation (layered cortical structure) for DLPFC sample 151507. (D) Spatial clustering result of AugGCL (ARI = 0.67) on sample 151507, showing clear identification of cortical layers. (E) UMAP visualization of AugGCL-learned embeddings on sample 151507. (F) Comparison of clustering results and UMAP visualizations from baseline models (DeepST, GraphST, SEDR, SpaGCN, stLearn, SiGra) on sample 151507.

To assess clustering accuracy, the widely adopted external clustering metric Adjusted Rand Index (ARI) was used. As shown in Fig 2A, AugGCL achieved the highest median ARI score among all compared methods, with the smallest interquartile range, demonstrating not only high accuracy but also exceptional stability across the heterogeneous tissue slices. In contrast, methods such as StLearn and SpaGCN exhibited broader ARI distributions and lower median scores, indicating that they are more sensitive to expression variability and structural complexity. These results emphasize that AugGCL is robust and generalizable in delineating spatial structures, consistently performing well across different tissue slices.

Further analysis is provided in Fig 2B, where the per-sample ARI scores of AugGCL across all 12 slices are shown. The results indicate that AugGCL achieved an ARI score greater than 0.5 for all slices, with most slices reaching scores close to or exceeding 0.6. This further validates AugGCL’s stable and reliable performance across various slice variations, emphasizing its robustness in different biological contexts.

To visually assess the spatial clustering capability of AugGCL, a representative sample from the DLPFC dataset (ID: 151507) was selected. As depicted in Fig 2C, the ground truth annotation clearly reveals the layered structure of the cortex, from Layer 1 to Layer 6, as well as the white matter. The spatial clustering result from AugGCL, shown in Fig 2D (ARI = 0.67), is highly consistent with this ground truth, presenting clear boundaries between layers and excellent structural continuity. This confirms that AugGCL effectively captures the complex spatial structures of the DLPFC tissue.

To further validate, the learned embeddings were visualized using UMAP, as shown in Fig 2E. The UMAP plot demonstrates well-separated clusters that align closely with the tissue structure annotations, indicating that AugGCL has successfully learned to represent the spatial features in the embedding space. In contrast, clustering results from baseline methods, presented in Fig 2F, exhibit varying degrees of segmentation noise, boundary blurring, and layer mixing. For instance, GraphST misclassifies several regions, while SpaGCN and StLearn exhibit over-segmentation at layer boundaries, further highlighting the superior performance of AugGCL in accurately reconstructing complex cortical structures with minimal distortion.

### Biological validation and analysis of spatial clustering results in AugGCL

**Fig 3:**
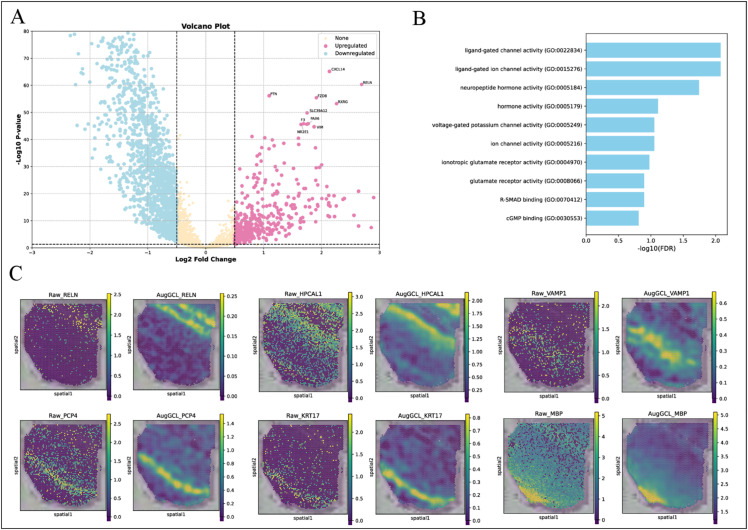
Biological evaluation of gene expression and clustering performance in DLPFC. (A) Volcano plot: The relationship between log2 fold change and -log10 p-value of differentially expressed genes in the DLPFC dataset. Genes are categorized as upregulated (pink), downregulated (blue), and non-significant (yellow). (B) GO enrichment analysis: The enrichment results of upregulated genes in DLPFC Layer 2, with the most enriched terms including chemical synaptic transmission and glutamate receptor signaling pathways. (C) Gene expression visualization: Visualization of key genes (RELN, HPCAL1, PCP4, KRT17, VAMP1, MBP) in both raw and enhanced formats. The enhanced images reveal clearer spatial expression patterns.

To better understand the biological significance of gene expression changes, differential expression analysis was first performed, and the results were visualized using a volcano plot (Fig 3A). This plot highlights several genes with significant expression changes, such as CXCL14 [31], RELN [32], PTN [33], and CNN3 [34], all of which show large fold changes and statistical significance. These findings suggest that these genes may play crucial roles in the human DLPFC and could serve as potential biomarkers for further investigation into brain function and disease.

Further Gene Ontology (GO) enrichment analysis showed that the upregulated genes in Layer 2 of the DLPFC are primarily involved in neurotransmitter signaling and synaptic activity. Notably, GO terms such as chemical synaptic transmission, anterograde trans-synaptic signaling, and regulation of glutamate receptor signaling pathways were highly enriched (Fig 3B). These results suggest that the upregulated genes in Layer 2 play a critical role in synaptic communication and the regulation of glutamate receptors, both of which are essential for the proper functioning of the DLPFC.

To visually assess gene expression in the tissue, the expression patterns of several key genes were examined using both raw and enhanced visualization methods (Fig 3C). While the raw expression maps displayed sparse and uneven distributions of gene activity, the enhanced maps provided clearer and more coherent visualizations, revealing distinct spatial patterns of gene expression. Specifically, genes such as PCP4 and KRT17 exhibited clear spatial clustering in the enhanced images, highlighting the spatial organization of gene activity in the DLPFC. The enhanced results enabled more precise identification and analysis of gene distribution across different regions of the DLPFC, offering deeper insights into the molecular architecture of this brain area.

With the enhancement provided by AugGCL, the spatial clustering and regional distribution of gene expression became much clearer, highlighting the significant advantages of this method in extracting and enhancing gene expression patterns in spatial transcriptomics. Compared to the raw images, the enhanced visualizations not only improved the clarity of gene activity but also revealed more accurately the spatial distribution and structural patterns of genes within the tissue.

### AugGCL for spatial domain recognition and biological evaluation in breast cancer dataset

In this study, AugGCL was applied to the spatial transcriptomics analysis of the human breast cancer dataset [35–38], assessing its performance in spatial domain recognition and clustering accuracy. Compared to several existing methods, AugGCL demonstrated distinct advantages in identifying spatial domains within tumor tissues.

**Fig 4:**
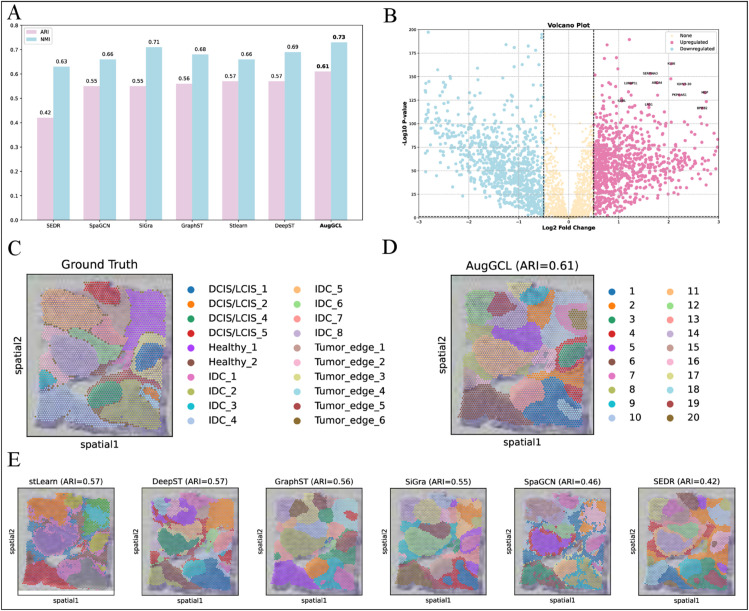
Model comparison and spatial clustering in breast cancer data. (A) Performance comparison of different models (stlearn, SpaGCN, SEDR, GraphST, DeepST, SiGra, and AugGCL) based on ARI and NMI scores. (B) Volcano plot showing the differential expression of genes, highlighting upregulated (pink) and downregulated (blue) genes based on log2 fold change and -log10 P-values. (C) Ground truth spatial clustering, with tissue regions labeled for DCIS/LCIS, IDC, healthy tissue, and tumor edges. (D) AugGCL clustering result (ARI=0.61) showing spatial clustering of tumor and healthy tissue regions. (E) Comparison of spatial clustering results from various models, including stLearn, DeepST, GraphST, SEDR, SpaGCN, and SiGra, with corresponding ARI values.

The performance of different models on the dataset was evaluated using two key metrics: ARI and Normalized Mutual Information (NMI) (Fig 4A). The results showed that AugGCL outperformed other models with ARI of 0.61 and NMI of 0.73, surpassing models such as stLearn (ARI = 0.57), SpaGCN (ARI = 0.55), and SiGra (ARI = 0.55). These results suggest that AugGCL not only identifies spatial domains more accurately but also delineates the boundaries between tumor and healthy tissue more effectively, demonstrating higher clustering precision. In contrast, other models exhibited lower ARI values, indicating their limited ability to distinguish spatial domains.

Next, the significantly upregulated and downregulated genes in the dataset were explored using a volcano plot analysis (Fig 4B). The volcano plot clearly highlights several key upregulated genes, such as SERPINA3 [39], KLK6 [40], and MGP [41], whose expression patterns are closely associated with different tumor subtypes, offering important insights for biomarker discovery and targeted therapies.

To better understand how these genes contribute to tumor progression, their functions and mechanisms were explored. SERPINA3, a serine protease inhibitor, may act as either a tumor promoter or suppressor depending on the tumor type, but its precise mechanisms remain unclear. KLK6 plays a crucial role in regulating tumor cell migration, invasion, and response to radiotherapy. Its elevated expression in invasive tumor regions is considered a key factor in tumor progression and metastasis. MGP is involved in tumor angiogenesis, regulating the maturation of blood vessels, affecting nutrient supply and tumor growth, and providing a potential therapeutic target for future treatment strategies.

In terms of spatial clustering, AugGCL effectively distinguished different tissue regions, including Invasive Ductal Carcinoma (IDC), healthy tissue, and tumor edges (Fig 4C). The clustering results from AugGCL were highly consistent with the ground truth labels (Fig 4D), particularly in clustering the tumor edge regions. This indicates that AugGCL is capable of effectively preserving spatial information and accurately reflecting the complex structure of the tumor microenvironment when processing spatial transcriptomics data.

Through comparison with other models (Fig 4E), the clear advantages of AugGCL were further validated. For instance, while GraphST (ARI = 0.56) and DeepST (ARI = 0.57) performed similarly, they still lagged significantly behind AugGCL. This further highlights the remarkable superiority of AugGCL in spatial data analysis, particularly in distinguishing tumor microenvironments from healthy tissues.

### Model performance evaluation of the E9.5 mouse embryo dataset

**Fig 5:**
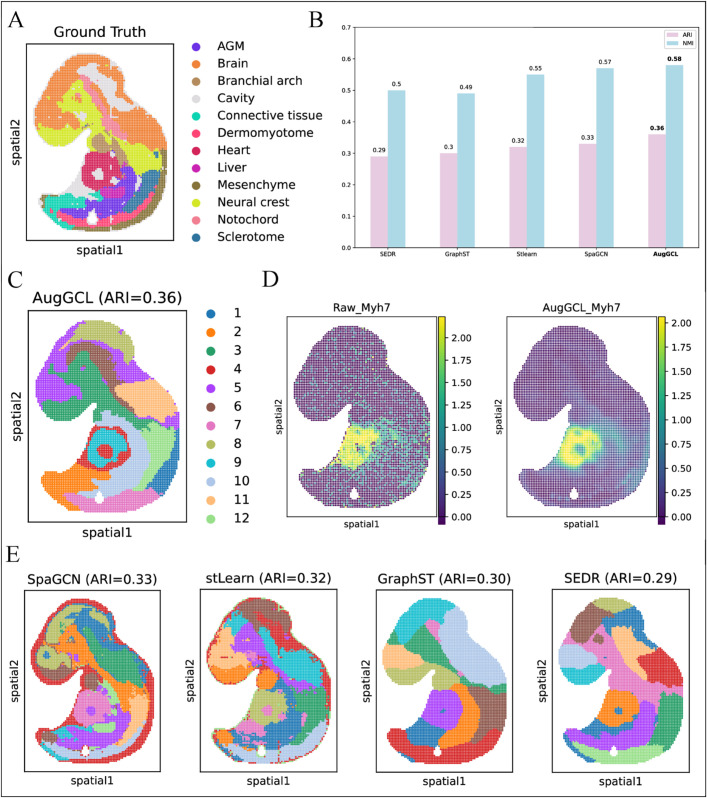
Decoding spatial patterns and clustering dynamics in the E9.5 mouse embryo transcriptome. (A) Ground Truth: The true labels for tissue regions in the mouse embryo E9.5 dataset, including key regions such as AGM, Brain, Heart, Liver, and Neural Crest. (B) Model Performance (ARI & NMI): Bar plot comparing the performance of different clustering models (stlearn, SpaGCN, SEDR, GraphST, AugGCL) based on ARI and NMI. (C) AugGCL Spatial Clustering Result: Spatial clustering results using AugGCL, with regions color-coded according to their predicted labels. (D) Gene Expression Analysis for Heart Marker Gene: Comparison of raw gene expression (Raw_Myh7) and AugGCL reconstructed gene expression (AugGCL_Myh7) for the heart marker gene Myh7. (E) Spatial Clustering Results Comparison: Spatial clustering results from different methods (SpaGCN, SEDR, stLearn, GraphST) with corresponding ARI values for each method.

This study applies spatial transcriptomic analysis to the Mouse Embryo E9.5 dataset [42] to explore gene expression patterns across various tissue regions during embryonic development. The dataset includes key developmental tissue regions, such as the aorta-gonad-mesonephros (AGM), brain, heart, liver, and neural crest (Fig 5A). These regions exhibit distinct spatial distributions and transcriptomic features, providing insights into cell fate determination and organ formation during early development.

The performance of different clustering models, including SpaGCN, SEDR, stLearn, GraphST, and AugGCL, was evaluated using the ARI and NMI (Fig 5B). AugGCL demonstrated superior precision in spatial domain identification and tissue differentiation, particularly in regions such as AGM, neural crest, and heart, with ARI of 0.36 and NMI of 0.58. In contrast, models like SEDR and GraphST showed relatively lower performance, revealing limitations in handling complex embryonic development data.

The spatial clustering results demonstrate that AugGCL effectively distinguishes key tissue regions, including the brain, heart, and neural crest, with strong alignment between the clustering results and ground truth labels (Fig 5C). This indicates that AugGCL accurately captures spatial tissue information, providing reliable data for further investigation of developmental processes.

Gene expression patterns for Myh7 were also compared between raw data and those reconstructed by AugGCL (Fig 5D). AugGCL demonstrated a clearer reconstruction of gene expression patterns, especially in the heart region.

A comparison of spatial clustering results from different methods (SpaGCN, SEDR, stLearn, GraphST) (Fig 5E) further confirms the superiority of AugGCL. It showed higher clustering accuracy and better retention of spatial structure between tissues compared to other methods.

## Discussion

Current spatial transcriptomics analysis methods still face considerable challenges in integrating multimodal information and improving the quality of spatial expression representations. In particular, when addressing challenges such as high gene expression sparsity, complex tissue architecture, and weak spatial signals, traditional approaches that rely on unimodal features or rule-based clustering often struggle to balance biological interpretability with structural resolution. To address these limitations, this study proposes the AugGCL framework, which aims to systematically enhance spatial structure decoding and expression pattern reconstruction through a neighborhood information aggregation mechanism and multimodal graph neural network modeling.

The key innovation of AugGCL lies in its integration of the neighborhood information aggregation module. This module jointly considers gene expression similarity and spatial proximity to dynamically generate a weighted graph structure, which is then used to perform spatial enhancement on the raw gene expression matrix. Compared to strategies that rely solely on spatial coordinates, neighborhood information aggregation incorporates functional-level regulatory signals, thereby improving both the consistency and discriminative power of cellular features. This is particularly advantageous in regions of sparse gene expression, effectively mitigating the expression void problem and enabling the construction of smoother, structure-aware expression representations for downstream modeling.

In terms of graph neural network modeling, AugGCL introduces a multimodal fusion architecture that integrates enhanced expression features with image-based morphological features as joint inputs. Through graph convolutional layers, the model unifies the encoding of spatial, functional, and morphological information of cells. The introduction of the image modality not only provides spatial boundary and morphological priors, but also enhances the clarity of cluster boundaries and continuity of tissue structures via auxiliary loss supervision. This multimodal fusion strategy enables AugGCL to effectively capture both local spatial variations and global structural partitions.

Experimental results demonstrate that AugGCL achieves superior performance across several representative ST datasets, including human cerebral cortex, breast cancer tissue, and embryonic development samples. In tasks such as identifying cortical layering, tumor boundary delineation, and cell lineage differentiation, AugGCL significantly outperforms mainstream baseline models, exhibiting strong robustness and generalizability. Furthermore, AugGCL introduces new approaches for gene expression reconstruction and spatial visualization. The enhanced expression maps it generates exhibit improved spatial coherence and biological relevance, providing a solid foundation for downstream functional annotation and mechanistic exploration.

In conclusion, AugGCL establishes a unified analytical framework based on multimodal collaborative modeling of expression, structure, and image data. By incorporating neighborhood-level information enhancement and image-guided fusion mechanisms, it significantly improves the interpretability of spatial expression data and the accuracy of tissue structure resolution. This makes AugGCL a generalizable and scalable computational tool for advancing spatial transcriptomics research.

## Materials and methods

### Data and image preprocessing

In the preprocessing phase, both the spatial transcriptomics data and corresponding tissue images were processed. First, the gene expression matrices were loaded from various platforms, along with their associated tissue images. A filtering process was applied to the gene expression data to remove low-expressing genes: (1) genes expressed in fewer than three cells were discarded to reduce noise; (2) genes known to introduce biases were excluded. The processed data was saved in .h5ad format for subsequent analysis, and the gene expression matrix was exported as a CSV file for further examination.

For the image preprocessing, the original histology image was read and converted to grayscale if necessary. To prepare the image for model input, it was then transformed into a tensor. Using the spatial coordinates (such as cell positions), image patches around each coordinate, typically of size 50*50 pixels, were extracted and flattened into one-dimensional vectors. All the extracted patches were stored in a dataframe, aligned with the original gene expression data’s index for easy access and further analysis. This combined preprocessing workflow ensures the retention of biologically relevant genes and image features, significantly enhancing the quality and reliability of the dataset for downstream analysis.

### Spatial graph construction

The construction of a spatial graph aims to better integrate information from neighboring cells, enhancing the relationships between the data points. By using spatial coordinates to compute the distances between cells and applying a predefined distance threshold or number of neighbors, it is possible to effectively capture the neighboring cells and their respective distance relationships.

Let $X=[x_{1},x_{2},\ldots ,x_{N}]$ represent the *N* cell sample data. Correspondingly, the matrix $X_{\text{r}}$ represents the raw gene expression data, the matrix $X_{\text{g}}$ represents the augmented gene expression data, and $X_{\text{i}}$ represents the tissue image data. *d*_*ij*_ is the distance between cells *x*_*i*_ and *x*_*j*_. The adjacency matrix *A* is determined as follows:

$$A_{ij}=\left\{ \begin{array}{cc} 1 & \text{if }d_{ij}\leq \text{threshold}\\ 0 & \text{if }d_{ij}>\text{threshold} \end{array}\right.$$ (1)

In Eq.(1), *d*_*ij*_ is selected as Euclidean distance of the spatial locations between two cells.

This equation illustrates that cell *i* and cell *j* are neighbors if their distance is less than or equal to a predefined threshold, and non-neighbors otherwise. Self-loops are removed to ensure that only valid neighbor relationships are considered. This adjustment helps maintain a graph where each node (cell) is connected to others based on proximity, which is essential for certain graph-based algorithms.

This process constructs a spatial relationship graph between cells, providing a quantitative perspective on cell-to-cell interactions and offering critical biological insights into the spatial distribution and interactions of cells within tissues.

### Neighborhood information aggregation

In spatial data analysis, data augmentation methods combine gene expression similarity with spatial neighborhood information between cells to generate richer expression data. Specifically, the method calculates the similarity between cells using the gene expression matrix and combines it with the spatial adjacency matrix to produce an enhanced expression matrix. This approach not only retains the spatial structural characteristics of the cells but also enhances the correlation of gene expression among neighboring cells. For ST data with morphological information, image segmentation is performed based on the spatial positions of each cell to extract the image features of cell. Using the same data augmentation method, a weighted spatial adjacency graph and edge weights are generated, thereby improving the ability of model to analyze complex spatial structures and providing more accurate data support for downstream tasks such as spatial clustering.

In this process, the similarity metric *S*_*ij*_ is used to measure the similarity between cells based on their gene expression and spatial neighborhood relationships. It can be expressed as:

$$S_{ij}=\exp(\alpha -d_{ij})-1,$$ (2)

where exp(·) is the exponential function, and α is a parameter. *d*_*ij*_ could be selected as cosine distance [43], and correspondingly, the α is set to be 2, as shown in Eq.(2).

The cosine distance *d*_*ij*_ reflects the similarity between the gene expression profiles of two cells. A smaller *d*_*ij*_ indicates higher similarity, which results in a larger *S*_*ij*_. This is because the exponential function exp(α − *d*_*ij*_) increases as *d*_*ij*_ decreases, emphasizing the similarity between cells. The higher the *d*_*ij*_ value, the more cells with similar expression patterns will be connected, resulting in stronger connections between cells. The visualization of this function has been added to Figure 1 in S1 Appendix, which appears in (1) Explanation of the Neighborhood Information Aggregation.

The aim of this step is to effectively integrate the spatial proximity relationship and gene expression similarity, and the integration method is as follows:

$$C_{ij}=A_{ij}\cdot S_{ij},$$ (3)

This weighting operation allows us to reflect gene expression relationships while incorporating spatial information, facilitating further analysis.

The normalization operation is performed on the weighted adjacency matrix *C* and the edge weights, as demonstrated below:

$${\hat{C}}_{ij}=\frac{C_{ij}}{\sum_{j}C_{ij}+\epsilon },$$ (4)

where ϵ is a small value (e.g., 10^−10^) used to avoid division by zero errors.

For the given gene expression matrix $X_{\text{r}}$, the enhancement aims to address the sparsity issue in gene expression data by combining spatial information from neighboring cells with gene expression similarities. The data is enhanced using the weighted adjacency matrix $\hat{C}$. The resulting matrix $X_{\text{g}}$ incorporates spatial neighbor information and improves the expression levels of the cells by leveraging the similarities of adjacent genes, as calculated below:

$$X_{\text{g}}=\beta \cdot \hat{C}\cdot X_{\text{r}}+X_{\text{r}},$$ (5)

where β=1.2 is a coefficient used to control the degree of enhancement.

### Graph neural network model designing

In this study, a novel graph convolutional network model with a two-layer architecture is proposed to enhance spatial data analysis by simultaneously processing both gene expression and tissue image features. The gene encoder processes the enhanced gene feature matrix $X_{\text{g}}$ with the gene adjacency matrix ${\hat{C}}_{g}$, while the image encoder processes the image feature matrix $X_{\text{i}}$, which represents the tissue image data, with the image adjacency matrix ${\hat{C}}_{i}$. Each encoder consists of 512 hidden units, with the ELU activation function applied after each convolution. The model is trained using a learning rate of 0.001 for 200 epochs, with a hidden dimension setting of [512, 30]. The model also integrates edge weights in a weighted multi-layer GCN structure to improve feature extraction capabilities, enabling effective decoding of complex spatial structures. After feature fusion, the model’s output is used for downstream spatial clustering tasks. These design choices enable the model to effectively process and integrate multimodal spatial data, while the chosen hyperparameters ensure optimal training for accurate clustering.

The model’s inputs include the enhanced gene feature matrix $X_{\text{g}}$, original gene expression matrix $X_{\text{r}}$, and image feature matrix $X_{\text{i}}$, which are associated with the weighted gene adjacency matrix ${\hat{C}}_{g}$ and the weighted image adjacency matrix ${\hat{C}}_{i}$, respectively. The forward propagation process for gene features can be represented as:

$$H_{g}^{l+1}=\sigma \left({\hat{C}}_{g}H_{g}^{l}W_{g}^{l}\right),$$ (6)

where $H_{g}^{l}$ is the feature matrix of the *l*-th layer for gene features, Additionally, $H_{g}^{0}$ is the enhanced gene expression matrix $X_{\text{g}}$. $W_{g}^{l}$ is the weight matrix for the *l*-th layer of gene features, and σ is the activation function.

The forward propagation process for image features can be represented as:

$$H_{i}^{l+1}=\sigma \left({\hat{C}}_{i}H_{i}^{l}W_{i}^{l}\right),$$ (7)

where $H_{i}^{l}$ is the feature matrix of the *l*-th layer for image features, Additionally, $H_{i}^{0}$ is the image expression matrix $X_{\text{i}}$. $W_{i}^{l}$ is the weight matrix for the *l*-th layer of image features.

After the individual feature propagation, the next step is to combine the gene and image features. By fusing $H_{i}^{2}$ and $H_{g}^{2}$, *H*_*c*_ is obtained,, which is then passed through a final fusion convolution layer to output $H_{c}^{2}$. The specific calculation process is as follows:

$$H_{c}=concat(H_{g}^{2},H_{i}^{2}),$$ (8)

$$H_{c}^{2}=\sigma \left(H_{c}W_{c}\right),$$ (9)

where *W*_*c*_ is the weight matrix for the fused features.

After further convolution and activation operations, the final output of the fused features $H_{c}^{2}$ will be used for downstream spatial clustering tasks. Additionally, the model supports the option to use image loss by setting parameters, allowing for optimization based on image features. This design enables the AugGCL model not only to retain the spatial structural characteristics of cells but also to enhance the correlation of gene expression among neighboring cells, providing more accurate support for spatial data analysis.

### Loss function construction

In this section, to help the model learn better, the overall optimization objective consists of multiple components. Reconstruction loss (*gloss*) calculates the difference between the original gene data and the reconstructed gene matrix, measured using Mean Squared Error (MSE). The goal is to minimize this difference, helping the model more accurately restore the gene data. Next, image reconstruction loss (*iloss*) measures the difference between the original image features and the reconstructed image matrix, ensuring that the spatial features of the image are well restored. Finally, regularization loss (*reloss*) calculates the difference between the fused reconstructed matrix and the original gene data, ensuring that the model can better fuse image and gene data while maintaining consistent feature representations. Note that the regularization loss is optional and can be used depending on whether it is necessary for the task. The specific calculation process is as follows:

$$gloss=\|X_{\text{r}}-{\text{Dec}}_{g}(H_{g}^{2}){\|}_{2}^{2},$$ (10)

$$iloss=\|X_{\text{i}}-{\text{Dec}}_{i}(H_{i}^{2}){\|}_{2}^{2},$$ (11)

$$reloss=\|X_{\text{r}}-{\text{Dec}}_{c}(H_{c}^{2}){\|}_{2}^{2},$$ (12)

where ${\text{Dec}}_{g}$, ${\text{Dec}}_{i}$, and ${\text{Dec}}_{c}$ represent the decoding operations for gene features, image features, and the fused features, respectively.

The final total loss is the weighted sum of these three components, with weights controlled by hyperparameters $\lambda_{1}$, $\lambda_{2}$, and $\lambda_{3}$. The formula is as follows:

$$loss=\lambda_{1}\cdot gloss+\lambda_{2}\cdot iloss+\lambda_{3}\cdot reloss,$$ (13)

By minimizing this total loss, the model can simultaneously optimize the reconstruction of gene data, image data, and the fused representation of both, thereby improving overall performance.

### Spatial clustering and visualization

In this study, two clustering methods, K-means and MCLUST, are employed to effectively identify complex spatial structures. The K-means clustering method, based on centroid optimization, efficiently detects cell types and their interactions, making it particularly suitable for large-scale datasets. On the other hand, MCLUST employs a Gaussian mixture model, enabling it to handle clusters of varying shapes and sizes, thus enhancing the interpretability of spatial data. Dimensionality reduction techniques, such as t-SNE or UMAP, are used to map high-dimensional data into lower-dimensional space, complemented by heatmaps and spatial distribution maps for visualization, thereby improving the understanding of cell distribution. By combining these two clustering methods with visualization techniques, an in-depth analysis of cellular spatial distribution can be conducted, specific cell subpopulations can be identified, and their variations under different biological conditions can be explored, providing crucial support for subsequent biomedical research.

To evaluate the clustering performance quantitatively, the adjusted rand index, a widely used external clustering metric, is employed [44–46]. The ARI assesses the agreement between the predicted clustering labels and the manually annotated ground truth while adjusting for chance. The ARI is calculated as follows:

ARI=∑ij(nij2)−[∑i(ai2)∑j(bj2)]/(n2)12[∑i(ai2)+∑j(bj2)]−[∑i(ai2)+∑j(bj2)]/(n2), (14)

The calculation of the ARI compares pairs of elements from the clustering result with pairs of cell types from the ground truth labels. By evaluating pairs of cells within the same cluster and the same real cell type, as well as between different clusters and different real cell types, ARI generates a value ranging from -1 to 1. A value of 1 signifies a perfect agreement between the predicted clusters and the true cell types.

Normalized mutual information measures the amount of shared information between two clustering results and is commonly used to assess the accuracy of unsupervised clustering methods. Given two sets of cluster labels *U* and *V*, NMI is defined as:

$$NMI(U,V)=\frac{MI(U,V)}{mean(K(U),K(V))},$$ (15)

where *MI* is the mutual information between *U* and *V*, and *K*(*U*), *K*(*V*) are the entropies of each label set. The formulas are as follows:

$$MI(U,V)=\sum_{i=1}^{\left|U\right|}\sum_{j=1}^{\left|V\right|}P(i,j)\log\left(\frac{P(i,j)}{P(i)P(j)}\right),$$ (16)

$$K(U)=-\sum_{i=1}^{\left|U\right|}P(i)\log P(i),$$ (17)

$$K(V)=-\sum_{j=1}^{\left|V\right|}P(j)\log P(j),$$ (18)

where *P*(*i*) is the probability of a sample belonging to the *i*-th cluster in *U*, and $P(j)=\frac{\left|V_{j}\right|}{N}$ is the probability of a sample belonging to the *j*-th cluster in *V*. $P(i,j)=\frac{n_{ij}}{N}$, where *n*_*ij*_ denotes the number of samples simultaneously assigned to cluster *U*_*i*_ and $V_{j}$, and *N* is the total number of samples. A NMI value closer to 1 indicates a higher level of agreement between the two clustering results.

### Ablation study and statistical significance analysis

The ablation study assesses the impact of different components of the AugGCL model, specifically gene modality, image modality, and multimodal fusion. The results show that combining both gene and image modalities consistently improves performance across all samples, highlighting the advantages of multimodal integration. The impact of the neighborhood information aggregation mechanism was also demonstrated, showing how the augmented gene matrix, after applying the mechanism, mitigates sparsity and enhances spatial domain identification. For more details, consult Figure 2 and Table 1 in S1 Appendix, which appear in (2) Ablation Study.

To compare the performance of AugGCL against other baseline models, statistical significance analysis was conducted using the independent sample t-test method. The results show that AugGCL consistently outperforms other models, with highly significant performance improvements. The p-values indicate that AugGCL achieves superior results compared to other models in most comparisons. The statistical performance metrics and p-value comparisons can be found in Table 2 and Table 3 in S1 Appendix, which appears in (3) Statistical Significance Analysis.

### Downstream analysis

In this section, downstream biological analyses [47] are performed to gain deeper insights into the clustering results and data processing. These analyses help uncover cellular and molecular characteristics. The gene expression heatmap offers a clear visualization of expression patterns across various tissue regions, enabling the identification of gene variability and its distribution among different cell types. The volcano plot compares log-fold changes in gene expression with statistical significance, helping to identify genes that are significantly upregulated or downregulated, and revealing cellular responses under various biological conditions. Gene Ontology enrichment analysis identifies biological processes, molecular functions, and cellular components that are significantly overrepresented among differentially expressed genes, providing further insight into the biological functions and regulatory mechanisms of the identified gene clusters. By combining these analyses, a comprehensive understanding of gene distribution in spatial contexts is achieved, functional differences across cell types are explored, and essential support is provided for subsequent biomedical research.

## Supporting information

S1 Appendix Supplementary materials. (1) Explanation of the Neighborhood Information Aggregation. (2) Ablation Study. (3) Statistical Significance Analysis.
(PDF)
