# Supplementary material for: AugGCL: Multimodal graph learning for spatial transcriptomics analysis with enhanced gene and morphological data
Source: PLoS Comput Biol. 2026 Jan 23;22(1):e1013912. doi: 10.1371/journal.pcbi.1013912 (PMC12863693; doi:10.1371/journal.pcbi.1013912)
Supplement: S1 Appendix — (1) Explanation of the Neighborhood Information Aggregation. (2) Ablation Study. (3) Statistical Significance Analysis. (PDF) [file pcbi.1013912.s001.pdf]

Supplementary information for 'AugGCL: Multimodal Graph Learning for Spatial Transcriptomics Analysis with Enhanced Gene and Morphological Data'

Tengfei Ji, Bo Yang*, Meng Wang, Hong Ji, Huazhe Yang, Yizhuo Liu
School of Computer Science, Xi'an Polytechnic University, Xi'an, Shaanxi, China
* yangboo@stu.xitu.edu.cn(BY);

Supplementary Notes

1. Explanation of the Neighborhood Information Aggregation

A detailed explanation of the S_{ij} similarity metric used in the Neighborhood Information Aggregation module is provided here. Specifically, the S_{ij} similarity metric is an exponentially decaying function of the distance d_{ij}, defined as:

$$S_{ij} = \exp\,(\alpha - d_{ij}) - 1$$

where d_{ij} represents the cosine distance between cells i and j, and α is a parameter that controls the decay rate. As the cosine distance d_{ij} increases, the similarity metric S_{ij} decreases exponentially, indicating that cells with more similar gene expression profiles are assigned higher similarity values.

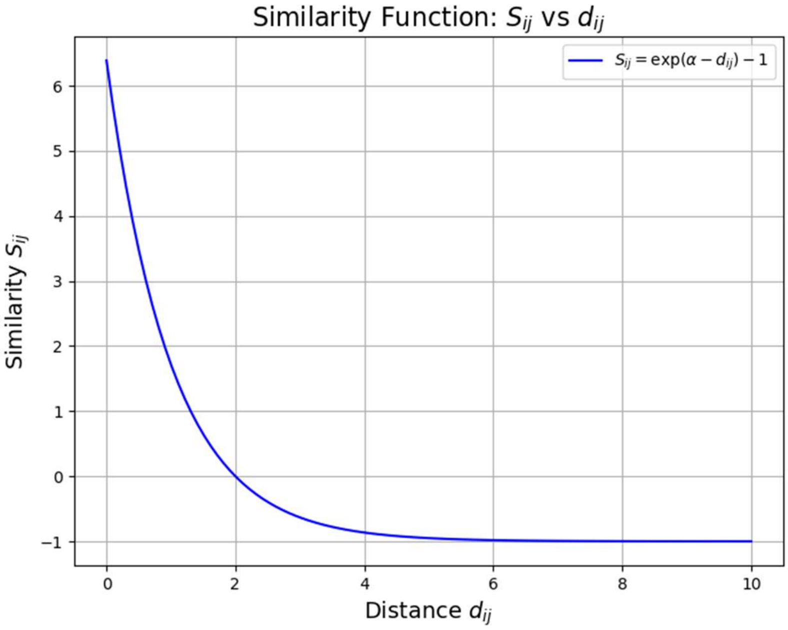

Figure 1: Similarity Function: S_{ij} vs Distance d_{ij}

To visually illustrate the relationship between d_{ij} and S_{ij}, an example graph (Figure 1) is provided. As shown in the figure, when d_{ij} is small, the similarity value S_{ij} is large, and as d_{ij} increases, S_{ij} gradually decreases. This visualization demonstrates how the similarity metric reflects the relationship between gene expression profiles.

2. Ablation Study for AugGCL Model

To assess the impact of different components in the AugGCL model, ablation studies were conducted. Specifically, a series of experiments were performed to compare the

performance of the model with and without key components, including gene modality, image modality, and multimodal fusion. The results of these comparisons are summarized in the table below, providing insights into how the inclusion of each component impacts performance. The evaluation metric used to measure performance is the Adjusted Rand Index (ARI), which is a commonly used metric to evaluate clustering results.

Table 1: Ablation Study Results

Sample	Img(iloss)	Gene(gloss)	Gene+Img(gloss+iloss)
151507	0.39	0.54	**0.67**
151508	0.41	0.50	**0.54**
151509	0.52	0.48	**0.60**
151510	0.42	0.57	**0.59**
151669	0.22	0.43	**0.56**
151670	0.26	0.37	**0.54**
151671	0.49	0.41	**0.65**
151672	0.39	0.44	**0.66**
151673	0.39	0.37	**0.61**
151674	0.36	0.36	**0.64**
151675	0.39	0.45	**0.64**
151676	0.42	0.31	**0.61**

As shown in the table above, combining both gene and image modalities (Gene+Img) consistently improves performance across all samples, demonstrating the advantage of multimodal integration. Additionally, for the Neighborhood Information Aggregation (NIA) mechanism, the effect of NIA was visualized by comparing the original gene matrix with the augmented gene matrix after applying NIA. As shown in the figures below, the augmented gene matrix, post-NIA, significantly mitigates sparsity, leading to improved spatial domain identification.

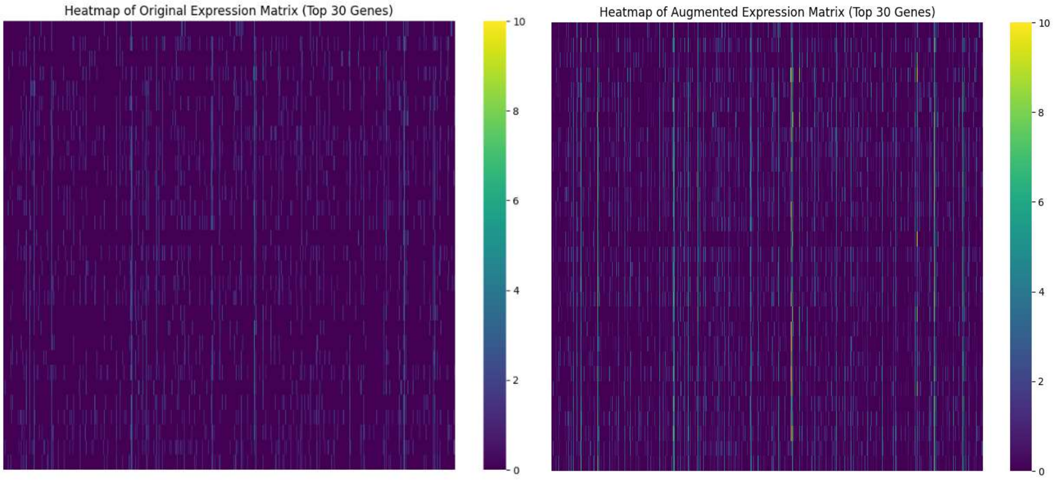

Figure 2: Comparison of Original and Augmented Gene Expression Matrices

In summary, the ablation studies demonstrate the significant impact of multimodal integration, particularly the combination of gene and image modalities, on improving model performance. The Neighborhood Information Aggregation mechanism effectively addresses sparsity and enhances spatial domain identification.

3. Statistical Significance Analysis of AugGCL Model

To further evaluate the performance differences between AugGCL and other baseline models, statistical significance analysis was conducted. Specifically, the independent sample t-test method was used to assess the performance differences between AugGCL and other models across different samples. The p-values were calculated, and the results are summarized below. The statistical significance analysis demonstrates that AugGCL shows significant performance improvements compared to most of the other models.

Table 2: Performance Summary of Different Models

Model	Mean	Std	Min	Max	Median
Stlearn	0.39	0.08	0.19	0.49	0.375
SpaGCN	0.42	0.09	0.23	0.56	0.435
SEDR	0.51	0.08	0.37	0.63	0.515
GraphST	0.55	0.08	0.38	0.63	0.585
DeepST	0.49	0.07	0.36	0.59	0.495
SiGra	0.42	0.10	0.23	0.56	0.42
AugGCL	**0.61**	**0.05**	**0.54**	**0.67**	**0.61**

Table 3: Statistical Significance Comparison

Model Comparison	p-value
AugGCL vs Stlearn	1.32e-08
AugGCL vs SpaGCN	1.33e-06
AugGCL vs SEDR	7.95e-04
AugGCL vs GraphST	4.02e-02
AugGCL vs DeepST	3.80e-05
AugGCL vs SiGra	5.00e-06

The p-values in the table 3 above show that AugGCL consistently outperforms other models, with highly significant performance improvements in most comparisons.
